# Effect of different electrostimulation currents on female urinary incontinence: A protocol of a randomized controlled trial

**DOI:** 10.1371/journal.pone.0276722

**Published:** 2022-12-01

**Authors:** Raissa Escandiusi Avramidis, Angélica Mércia Pascon Barbosa, Guilherme Thomaz de Aquino Nava, Danielle Hikaru Nagami, Caroline Baldini Prudencio, Cristiane Rodrigues Pedroni

**Affiliations:** 1 Department of Physical Education, Institute of Biosciences of Rio Claro, São Paulo State University (UNESP), Rio Claro, São Paulo, Brazil; 2 São Paulo State University (Unesp), Postgraduate Program on Tocogynecology, Botucatu Medical School, Botucatu, Brazil; 3 São Paulo State University (Unesp), School of Philosophy and Sciences, Marilia, Brazil; UNITED STATES

## Abstract

**Introduction:**

Urgency urinary incontinence (UUI) is characterized by involuntary urine leakage immediately after reporting of sudden, compelling desire to void. Electrostimulation and non-invasive neuromodulation have been considered as the first and third line of UUI treatment but there is a lack of consensus on which parameters are more efficient. Thus, this study aims to compare the effect of low versus medium frequency currents on urinary incontinence severity and quality of life in women with UUI complains.

**Methods:**

It will be a randomized controlled trial with 5 arms, double-blinded (outcome assessor and statistician). The study was approved by the Research Ethics Committee (CAAE: 11479119.9.0000.5406) and has been prospectively registered on the Brazilian Registry of Clinical Trials (RBR-8bkkp6). Concerning, double-blind process, the blinded assessor will be responsible for evaluate primary and secondary outcomes at baseline and follow-up without information about allocation and the statistician will perform analyses without information about group codification. One hundred and five participants will be randomized to receive: (1) Transcutaneous tibial nerve stimulation-low frequency, (2) Transcutaneous tibial nerve stimulation-high frequency, (3) Aussie median frequency, (4) Interferencial median frequency or (5) High voltage stimulation. The application will be performed during 20 sessions of 45-minutes, twice a week for 10 weeks, in groups of maximum 5 participants. The participants will be evaluated before treatment (baseline- 0 week), during the treatment (5 weeks) and after the last treatment session (10 weeks). The primary outcomes measures will be UI severity and quality of life, and the secondary outcome will be pelvic floor strength. Statistical analysis will be performed using SPSS software version 24.0 for Windows (IBM Corp., Armonk, N.Y., USA). The variables will be described by the mean and 95% confidence interval. The distribution of normality will be analyzed by the Shapiro-Wilk test. ANOVA for repeated measures will be performed. Mauchly’s test the hypothesis of sphericity and when if this violated the hypotheses, the analyses will be based on the Greenhouse-Geisser test. Peer-to-peer comparisons will be performed using the Bonferroni Post-Hoc test. The significant level adopted will be 5% (p ≤ 0.05).

**Conclusion:**

This study will enhance knowledge about effect of different neuromodulation currents in the improvement of UUI.

## Background

Urinary incontinence (UI) is a public health problem and affects millions of women worldwide [1]. Defined as involuntary loss of urine [2] UI negatively impact social, economic, familiar, and sexual life aspects [3, 4]. Urgent urinary incontinence (UUI) is characterized by involuntary urine leakage associated with urgency [2] and, in addition to the negative impacts already mentioned, it also causes embarrassment [4]. The prevalence of UUI varies between 5 to 50% of the world population and may increase in elderly population [5].

Although the electrostimulation of pelvic floor muscles (PFM) has been considered as the first line of treatment for UUI [1], neuromodulation delivery by transcutaneous electric nerve stimulation (TENS) via tibial nerve is widely used in clinical settings for being easy to handle, has good acceptance and offering minimal contraindications and collateral effects [5]. TENS can be performed by placing electrodes in the suprapubic, perineal, tibial, or sacral region, however, the application in the region of the tibial nerve is undoubtedly the most used [5]. The heterogeneity on reporting of duration, frequency, work cycle, current, form of administration, and type of electrodes to manage UUI, restrict the knowledge of its effects on bladder modulation due to potential different mechanisms related to doses applied.

The most therapeutic neuromodulation TENS parameters used for urgency symptoms treatment are low pulse width (200 μs) and low frequency (10 Hz) [6], but other therapeutics possibilities have being studied and showed their beneficial effects [7]. Medium frequency currents, such as Aussie and Interferential, are modulated to offer the effect of low frequency current with the benefit of reduced unpleasant effects, such as pain or skin irritation [6].

The Aussie current has been suggested as an option due to its comfort during application, as well as reducing the impedance of the skin ensuring that the pulse of the current reaches deeper tissues [8]. The Interferential current is based on two medium frequency currents that interfere between each other and produce a low frequency resultant current. Its main effects are the significant increase in the pain threshold, which implies more current pleasant sensation [9] with the benefit of reaching deeper tissues [10]. The High Voltage current is also single-phase and pulsed, with double peak and reaches sensory and motor nerve fibers responsible for nociceptive impulses, in addition, when modulated to low frequencies produce a pleasant sensation [11].

Despite the neuromodulation options described in the literature for improving UUI, the evidence is not conclusive, mainly because is still not clear whether the improvement in symptoms occurs due to the application of a specific type of electric current or if the parameters applied are responsible for the improvement. Therefore, the objective of the study will be to compare the effects of applying low frequency currents versus medium frequency currents on severity and quality of life at before treatment (baseline- 0 week), during the treatment (5 weeks) and after the last treatment session (10 weeks) in women with UUI. Our hypothesis is that medium frequency currents may be more effective in UUI treatment when compared to low frequency currents, considering that higher frequencies reduces skin’s impedance which implies delivery of the electrical current to deeper tissues and promotes more assertive stimulation to the tibial nerve which is involved on bladder activity modulation.

## Material and methods

### Design

**Fig 1:**
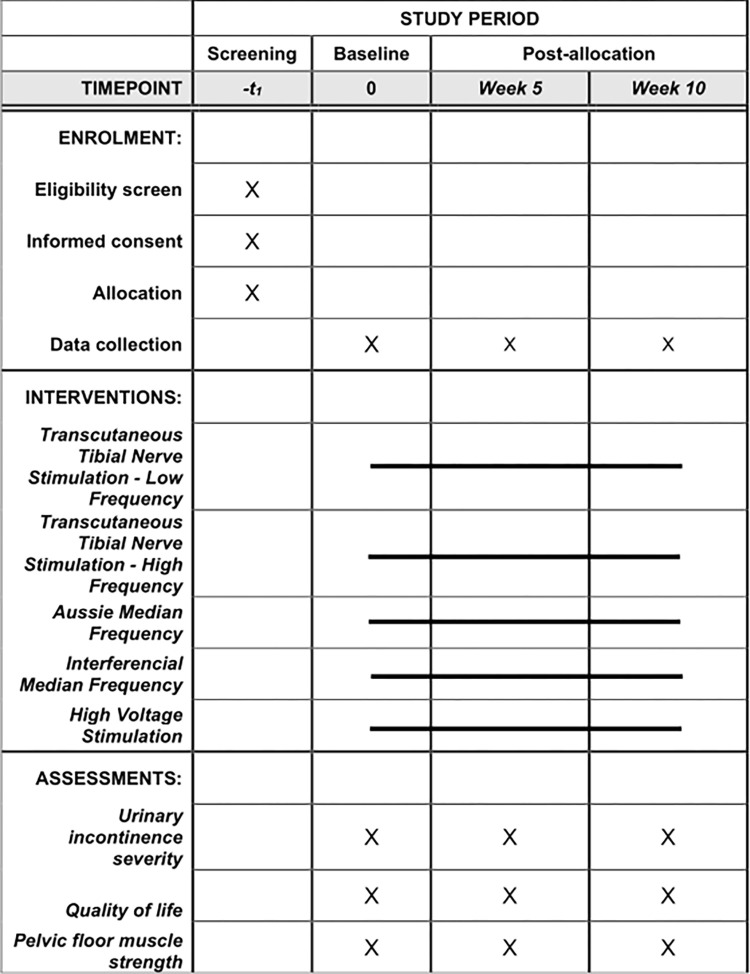
Schedule of the study protocol according to the standard protocol items: Recommendations for interventional trials checklist.

**Fig 2:**
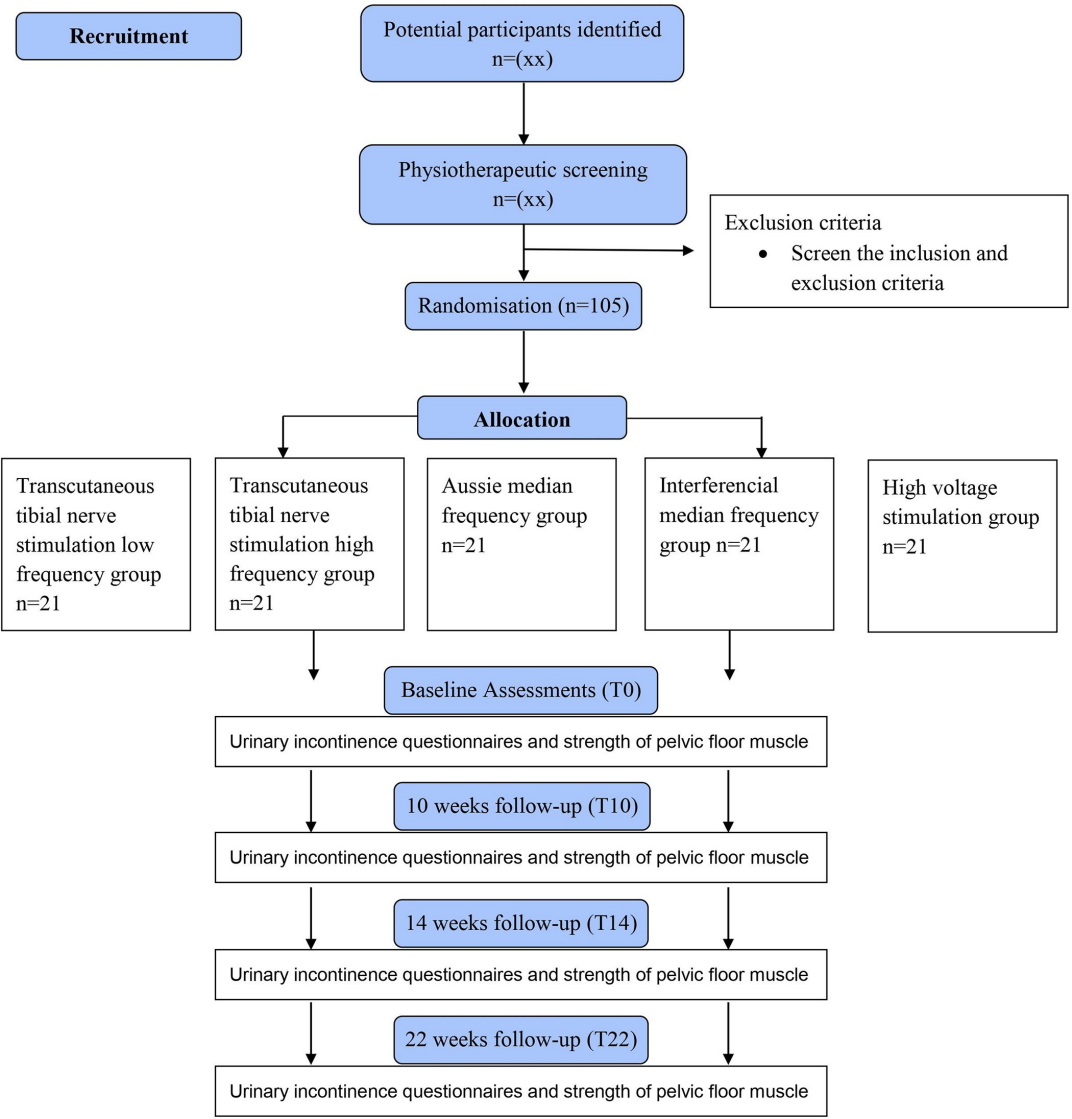
Consort flowchart.

A five-arm, parallel randomized controlled trial, double-blinded (outcome assessor and statistician), will be performed. All personal data will be confidential. The study follows the TIDieR (Template for Intervention Description and Replication) checklist [12], the 2013 Standard Protocol Items: Recommendations for International Trials statement [13] (Fig 1), and the Consort guidelines [14] (Fig 2).

### Ethical approval and consent

The study was approved by the Research Ethics Committee (CAAE: 11479119.9.0000.5406). All participants will be informed about the research and will sign a consent form. The protocol of this study has been prospectively registered on the Brazilian Registry of Clinical Trials (RBR-8bkkp6). Participants will be informed about all study procedures and asked to sign the Informed Consent Form prior to their enrollment in the study.

### Participants

#### Recruitment

Women with UUI will be recruited from a waiting list from the Specialized Center for Women’s Health Rehabilitation of the São Paulo State University, Marília, São Paulo, Brazil.

### Eligibility criteria

#### Inclusion and exclusion criteria

Potential participants will be eligible for the study if they are between 18 to 80 years old with complaints of UUI. Potential participants will be ineligible for the study if they have cognitive impairment, psychiatric disorders, pregnancy, diagnosis of neurogenic bladder, pelvic organ prolapse greater than grade II, ongoing signs and symptoms of urinary tract infections, use of anticholinergic drugs, antagonists calcium, beta-antagonists, and dopamine antagonists, cannot commit to participating for the duration of treatment and those who do not sign the consent form.

### Clinical assessment

#### Physiotherapeutic evaluation

Potential participants who fulfil the eligibility criteria undergo baseline assessments. The protocol will include anamnesis and physical assessment, the clinical screening will be carried out at São Paulo State University and will take approximately 30 minutes.

A registered physiotherapist with 8 years of experience on urogynecological examination, and who has a Master in Gynecology and Obstetrics field supervised by physiotherapist that has more than 30 years of urogynecological assessment experience will perform the evaluation. Both assessors will not be enrolled on the screening, randomization and/or treatment routine to ensure blinding assessment regardless the treatment type.

The first step of data collection will be related to anthropometric data (age, weight, height, and BMI), personal information, demographic partner (profession, race and education) and pathological and clinical history. The personal data of the participants will be numerically coded and stored in a database.

The second step of data collected will be the physical examination. PFM function will be assessed through the PERFECT scheme [15] and will be held in a reserved and adequate room. In addition, they will answer four different questionnaires regarding UUI. The International Consultation on Incontinence Questionnaire- short form (ICIQ-SF) [16], the Overactive Bladder Questionnaire (OAB-V8) [17], the International Consultation on Incontinence Questionnaire Overactive Bladder (ICIQ-OAB) [18] and the Incontinence Severity Index (ISI) [19]. At the end of the baseline assessment and before the first treatment session, participants will receive the voiding diary and instructions to complete it at home during 3 different days. All questionnaires, including the voiding diary, will be administered in Portuguese (Brazil) according to their respective validated versions.

The same blinded assessor will evaluate the participants before treatment (baseline- 0 week), during the treatment (5 weeks) and after the last treatment session (10 weeks).

### Sample size and power analysis

A priori sample size calculation was performed using the G* Power 3.1 software. As the innovative comparisons methodology of this trial did not allow us to state our sample calculation on literature data, we calculated our sample size, based on F tests, ANOVA: repeated measures, within-between interactions, an effect size f of 0.17 were calculated considering a direct method using a small partial eta square (*n*^2^) of 0.03. Considering that we selected 5 therapies with potential treatment effects and we expect minimal differences between them, we considered the small effect size calculated by this appropriated method. In addition, we selected a power of 0.85, α error probability of 0.05, number of groups 5, number of measures 3, correlation among repeated measures of 0.5 and nonsphericity correction of 1 and a 10% dropout rate which estimated the recruitment of 105 participants, 21 per group.

### Randomization, allocation and blinding

If all inclusion criteria are fulfilled and if the presence of UUI is determined, the participants will be randomly assigned to receive: (1) Transcutaneous Tibial Nerve Stimulation—Low Frequency (TTNS–LF), (2) Transcutaneous Tibial Nerve Stimulation—High Frequency (TTNS–HF), (3) Aussie Median Frequency (AMF), (4) Interferencial Median Frequency (IMF) and (5) High Voltage Stimulation (HVS). To ensure homogeneous distribution between groups, the participants will be randomly allocated to five therapeutic arms using permuted, block-randomization to balance the number of participants allocated to each group. The permuted block (with 15 participants per block) randomization sequence will be done through the website www.sealedenvelope.com by another physiotherapist unrelated to assessment process. After the assessment and randomization the participants will be schedule to the treatment sessions and will be informed about which group was allocated, but the difference between the groups will not be mentioned. All participants will be advised not to disclose to the assessor any details about the intervention program that they have received (to ensure that allocation is concealed from the assessor).

### Interventions

The intervention will be performed during 20 sessions of 45-minutes, twice a week for 10 weeks. All participants will carry out the treatment in groups of maximum 5 participants in the same room. They will remain seated comfortably during the entire treatment session. All participants will have their tibial nerve stimulated, with the current intensity adjusted to the highest possible sensory level, modified according to their habituation to the current, without reaching the motor threshold or causing pain [21].

### Currents

○ **TTNS-LF:** Symmetrical biphasic pulsed current with 10Hz frequency, a pulse width of 200μs [6]. (Neurodyn Portable Ibramed^®^)
○ **TTNS-HF:** Symmetrical biphasic pulsed current with 150Hz frequency, 100μs pulse width [10] (Neurodyn Portable Ibramed^®^).
○ **AMF:** Medium frequency sinusoidal alternating current, with 4kHz carrier frequency and 4ms bursts with 100Hz frequency [20] (Aussie Sport Ibramed^®^).
○ **IMF:** Biphasic pulsed current of medium frequency, with 4kHz with a modulated frequency of 100Hz and a with sweep of 10Hz [10] (Neurovector Ibramed^®^).
○ **HVS**: Single-phase pulsed current, with triangular (exponential) twin pulses, frequency of 100Hz [11]. For this study, cathodic stimulation (negative pole) will be used (Neurodyn Portable Ibramed®).

### Electrode placement

**Fig 3:**
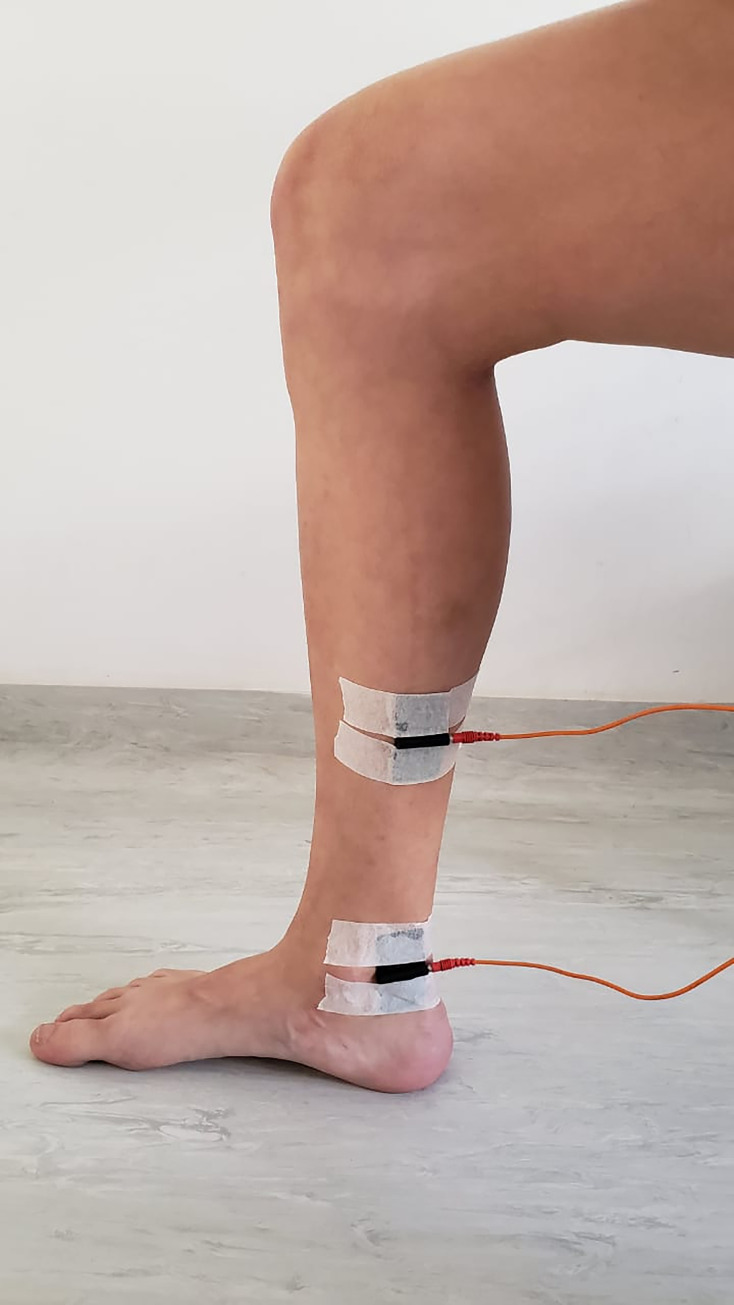
Electrode placement.

For all groups, the current will be applied through a channel with two electrodes, one positioned posterior to the medial malleolus and the other 10 cm above. Uniform and rectangular silicone-carbon electrodes with 6x4.5 centimeters will be used. In addition, water-based gel will be applied as interface with the skin (Fig 3) [21].

**Fig 4:**
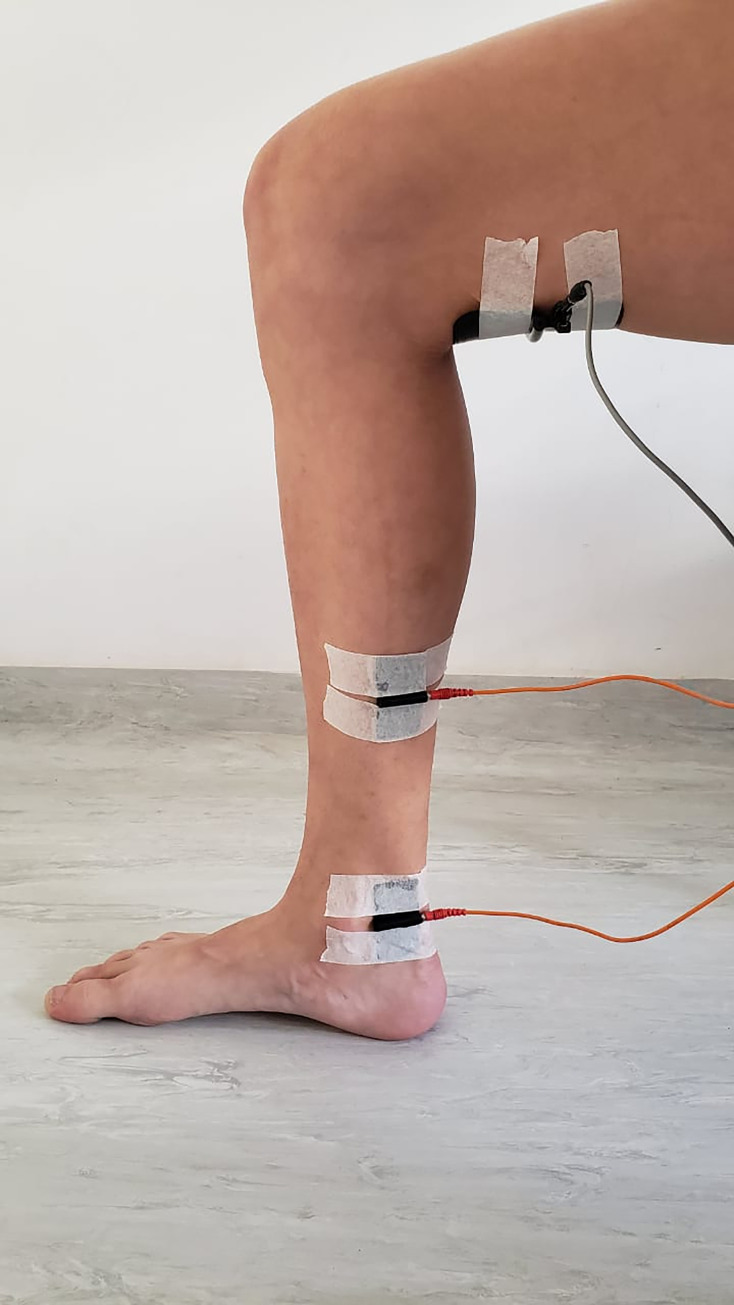
Positioning of the dispersive electrode.

Before placing the electrodes, the skin will be cleaned with 70% alcohol. All groups will receive transcutaneous electrical stimulation through the tibial nerve for 30 minutes. However, on the high voltage group a third dispersive electrode of 13x9 centimeters will be positioned above the popliteal fossa to ensure the direction of the electrical current from tibial nerve directed to the sciatic nerve (Fig 4).

### Outcome measures

#### Primary outcome measure

The primary outcomes will be: 1) UI severity and 2) quality of life.

1) ISI is a validated questionnaire to Portuguese, that classify UI according to severity by multiplying two simple and brief questions about incontinence frequency and volume. The severity score is stratified into mild (one to two points), moderate (three to six points), severe (eight to nine), or very severe (total of 12 points) [19]. The ISI questionnaire will be applied in all time-points.
1) Voiding diary is a self-real-life-assessment of voiding habits [22]. The 24-hour voiding diary shows greater acceptance by patients, but three-day voiding diary seems to be more suitable for possible evaluative interpretations [3]. The participants will fulfill during entire 3-days (72h) detailed information (including time of the event) about fluid intake (quantity), voiding (volume for each), leakage (time and associated reasons) and pad changes [22]. The voiding diary will be applied in all time-points.
2) The ICIQ-SF is a brief self-administered questionnaire combined severity and bother-score through 4 questions about incontinence frequency, volume, impact of UI in daily life and self-diagnosis questions about mechanism reasons. The higher the score obtained is, the worse the quality of life related to that domain is [16]. The ICIQ-SF questionnaire will be applied in all time-points.
2) The OAB-V8 will be used to assess the impact of overactivity bladder symptoms on quality of life. It is a questionnaire composed of 8 questions about the discomfort caused by the symptoms. The responses range from 0 to 5 points and in the sum of the results, if the total is ≥ 8, it will be considered as a probable diagnosis for UUI [17]. The OAB-V8 questionnaire will be applied in all time-points.
2) The ICIQ-OAB questionnaire provides an assessment measure for voiding frequency, urgency, nocturia, and UI. It is composed of six specific questions about voiding symptoms correlating with quality of life, and the higher the score the worst the impairment [18]. The ICIQ-OAB questionnaire will be applied in all time-points.

### Secondary outcome measure

The study will have one secondary outcome: 1) Assessment of the strength of the pelvic floor muscles (PFM) by PERFECT scheme.

The PFM assessment will be held before treatment (baseline- 0 week), and after the last treatment session (10 weeks). The first step bladder emptying will be requested to participant. After, the participant will be instructed to lay-down in supine position, with the lower limbs flexed, and the feet on the stretcher [15]. Prior to the PFM assessment explanation about the sequence of the PERFECT scheme will be done. Three domains of PERFECT will be used: Power, Endurance and Fast. Power (P) will be measured by Modified Oxford scale [23], which is a subjective measurement of the maximum voluntary contraction (MVC) from 0–5. We will considered the PFM strength as: 0 = no contraction- absence of muscle contraction, 1 = flicker contraction- outline of tremulous contraction (not sustained), 2 = weak contraction (which is sustained with little intensity), 3 = moderate contraction (slight lift of the examiners fingers without cranial movement), 4 = good contraction (elevation of the vaginal wall with compression of the examiner’s fingers towards to the pubic bone) and 5 = strong contraction (stronger compression of the examiner’s fingers towards the pubic bone combined with cranial movement) [24]. After strength classification, Endurance (E), referring to muscle resistance, will be assessed identifying duration of contraction up to 10 seconds using a chronometer. Fast (F) consists to the ability to perform up to 10 flicks contractions, the participant will be asked to perform a sequence of 10 MVC and the number of effective contractions will be recorded [25].

### Statistical analysis

The study will be run as a superiority trial. The statistical analysis will follow the per-treatment concept. Statistical analysis will be performed using SPSS software version 24.0 for Windows (IBM Corp., Armonk, N.Y., USA). The variables will be described by the mean and 95% confidence interval (95% CI). The distribution of normality will be analysed by the Shapiro-Wilk test. To analyse the effect of group and currents, ANOVA for repeated measures will be performed. The Mauchly’s test will be performed to test the hypothesis of sphericity and when if this violated the hypotheses, the analyses will be based on the Greenhouse-Geisser test. Peer-to-peer comparisons will be performed using the Bonferroni Post-Hoc test. The significant level adopted will be 5% (p ≤ 0.05).

## Discussion

This study was designed to compare the use of low and medium frequency currents by transcutaneous electrical nerve stimulation for UUI treatment. Concerning the instruments that will be applied during time-points assessments, the questionnaires are widely used to screening and diagnosis of UI and more over to quantify severity and impact on quality of life [19].

The use of TTNS–LF current is common in the literature and widely used in clinical practice to treat urgency related symptoms [26], although its mechanism is not yet widely known. The most accepted theory is that neuromodulation through the tibial nerve, which has nerve roots where parasympathetic fibers of the bladder originate, causes somatic depolarization of afferent fibers and generates inhibition of bladder activity [26].

Medium frequency currents have the advantage of producing smaller pulses during a period, reducing the impedance of the skin, which can generate greater current absorption through deeper tissues, besides, offer more comfortable sensation [6, 27]. Studies are limited to explore effects of different currents and doses options to promote neuromodulation [6]. Thus, it is necessary to enhance knowledge on this field.

The currents that will be used in the study were chosen due to their wide use in clinical practice in several areas of physiotherapy, moreover, to the characteristics of the currents [21] and the fact that they have similar results in other clinical situations [10]. We hypothesized that TTNS-LF may be more advantageous than the TTNS-HF, since the difference adopted between frequency and pulse width may guarantee greater dose [28]. In addition, the AMF current has the burst option, which divides the frequency pulses, which contributes to absorbing more current through the skin and should be an advantage over the others [20].

The IMF differential is the production of two medium frequency currents that interfere with each other, which allows the tissue to absorb more deeply [10] and can be beneficial for tibial nerve stimulation. The HVS has a third dispersive electrode and modulated at low frequency, thus ensuring greater comfort, and generating greater absorption [11].

In order to maintain the highest level of methodological quality, this RCT will be based on the CONSORT checklist. Participants will be randomized by a blinded assessor and will not receive information about the differences between the treatments offered. The assessors and statistician will not have contact with the participants during the intervention period and will not have knowledge about the group composition.

This study arose from the intention to cover the knowledge about electrotherapy for UUI treatment. Stimulation of the tibial nerve with TTNS is a treatment with favorable results, but many gaps on the knowledge remains [7].

### Dissemination

The study results will be circulated to peer-reviewed journals, lectures and scientific meetings.

## Conclusion

This study will help determine which parameters are more efficient in electrical stimulation for the improvement of UUI.

## Supporting information

S1 Checklist SPIRIT 2013 checklist: Recommended items to address in a clinical trial protocol and related documents*. (DOC)

S1 Protocol (PDF)

S2 Protocol (PDF)

S1 Appendix (DOCX)
