# Supplementary material for: Effect of different electrostimulation currents on female urinary incontinence: A protocol of a randomized controlled trial
Source: PLoS One. 2022 Dec 1;17(12):e0276722. doi: 10.1371/journal.pone.0276722 (PMC9714840; doi:10.1371/journal.pone.0276722)
Supplement: S1 Protocol — (PDF) [file pone.0276722.s002.pdf]

PARECER CONSUBSTANCIADO DO CEP

DADOS DO PROJETO DE PESQUISA

Título da Pesquisa: Efeito de diferentes correntes de eletroestimulação no nervo tibial em mulheres com incontinência urinária: Ensaio Clínico Randomizado

Pesquisador: CRISTIANE RODRIGUES PEDRONI

Área Temática:

Versão: 1

CAAE: 11479119.9.0000.5406

Instituição Proponente: Faculdade de Filosofia e Ciências/ UNESP - Campus de Marília

Patrocinador Principal: Financiamento Próprio
FUNDACAO DE AMPARO A PESQUISA DO ESTADO DE SAO PAULO

DADOS DO PARECER

Número do Parecer: 3.272.572

Apresentação do Projeto:

INTRODUÇÃO: A Incontinência Urinária é definida como queixa de qualquer perda involuntária de urina. Pode ser classificada em três principais tipos, a de esforço, a de urgência e a mista. A Incontinência urinária de urgência caracteriza-se por perda involuntária de urina associada à urgência miccional, policiúria e noctúria. Alguns tipos de eletroestimulação de baixa frequência têm sido utilizadas na prática clínica relacionada ao tratamento da incontinência urinária de urgência, porém ainda não se sabe se as correntes de média frequência podem ter efeitos semelhantes aos das correntes de baixa frequência. OBJETIVO: O objetivo do estudo será verificar os efeitos da aplicação das correntes de média frequência em mulheres que apresentam incontinência urinária de urgência e comparar os efeitos com os das correntes de baixa frequência. MÉTODO: Participarão desta pesquisa 105 voluntárias com idades entre 18 e 80 anos, que apresentarem queixas de hiperatividade vesical (frequência urinária aumentada, nocturia ou urge/incontinência. Serão aplicados os questionários ICIQ-SF, ICIQ-OAB, OAB-V8 e ISI, e também o diário miccional para avaliar os sintomas da hiperatividade vesical. Serão compostos 5 grupos, que serão nomeados de acordo com o tipo de corrente que será aplicada: G-TENS Uroginecológico e G-TENS Convencional serão tratados com TENS, G-Ausie receberá corrente Ausie, G-Interferencial receberá a corrente interferencial e o G-Alta Voltagem. Todas as voluntárias realizarão tratamento com eletroestimulação entre duas a três vezes por semana, totalizando 20

Endereço: Av. Hygino Muzzi Filho, 737
Bairro: Campus Universitário **CEP:** 17.525-900
UF: SP **Município:** MARILIA
Telefone: (14)3402-1346 **E-mail:** cep.marilia@unesp.br

sessões de 30 minutos cada. O processamento estatístico será realizado por meio do software SPSS, versão 18.0 (SPSS Inc, Chigaco, IL). Os dados serão apresentados em médias e intervalo de confiança a 95% para cada variável. O teste de Shapiro-Wilk será utilizado para analisar a normalidade dos dados. Para distribuição normal serão utilizados os testes paramétricos para comparação das médias, se não, serão utilizados seus respectivos testes não-paramétricos.

Objetivo da Pesquisa:

Objetivo Geral

O objetivo do estudo será verificar os efeitos da aplicação das correntes de média frequência em mulheres que apresentam incontinência urinária de urgência.

Objetivos Específicos

Verificar se a estimulação com corrente elétrica de média frequência produz efeitos benéficos no sentido de diminuir a frequência e o volume de perda urinária em mulheres com incontinência urinária de urgência, bem como melhorar a qualidade de vida dessas mulheres.

Comparar os efeitos das correntes de média frequência com os efeitos das correntes de baixa frequência utilizadas para o tratamento da incontinência urinária de urgência.

Avaliação dos Riscos e Benefícios:

Não se aplica.Não terá ônus para o participante e o mesmo receberá atendimento, se necessário, e orientações que lhe ajudarão em sua saúde.

Comentários e Considerações sobre a Pesquisa:

A pesquisa encontra-se dentro dos critérios éticos.

Considerações sobre os Termos de apresentação obrigatória:

Foram apresentados e analisados os termos solicitados pelo Comitê de ética em Pesquisa com seres humanos.

Conclusões ou Pendências e Lista de Inadequações:

Aprovado.

Considerações Finais a critério do CEP:

O CEP da FFC da UNESP de MARÍLIA, em reunião ordinária de 17/04/2019, após acatar o parecer do membro relator previamente aprovado para o presente estudo e atendendo a todos os dispositivos das resoluções 466/2012, 510/2016 e complementares, bem como ter aprovado o Termo de Consentimento Livre e Esclarecido como também todos os anexos incluídos na pesquisa,

Endereço: Av. Hygino Muzzi Filho, 737
Bairro: Campus Universitário **CEP:** 17.525-900
UF: SP **Município:** MARILIA
Telefone: (14)3402-1346 **E-mail:** cep.marilia@unesp.br

resolve APROVAR o projeto de pesquisa Efeito de diferentes correntes de eletroestimulação no nervo tibial em mulheres com incontinência urinária: Ensaio Clínico Randomizado

Este parecer foi elaborado baseado nos documentos abaixo relacionados:

Tipo Documento	Arquivo	Postagem	Autor	Situação
Informações Básicas do Projeto	PB_INFORMAÇÕES_BÁSICAS_DO_PROJETO_1313429.pdf	09/04/2019 11:51:49		Aceito
Cronograma	Cronograma_ECR_corrigido.docx	09/04/2019 11:51:30	CRISTIANE RODRIGUES PEDRONI	Aceito
Projeto Detalhado / Brochura Investigador	projeto_pesquisa_corrigido.doc	09/04/2019 11:50:07	CRISTIANE RODRIGUES PEDRONI	Aceito
Declaração de Instituição e Infraestrutura	declaracao.pdf	29/03/2019 16:08:22	RAISSA ESCANDIUSI AVRAMIDIS	Aceito
TCLE / Termos de Assentimento / Justificativa de Ausência	tcle.pdf	19/03/2019 21:03:15	RAISSA ESCANDIUSI AVRAMIDIS	Aceito
Folha de Rosto	folha_rosto.pdf	19/03/2019 21:01:15	RAISSA ESCANDIUSI	Aceito

Situação do Parecer:
Aprovado

Necessita Apreciação da CONEP:
Não

MARILIA, 18 de Abril de 2019

Assinado por:
CLAUDIO ROBERTO BROCANELLI
(Coordenador(a))

Endereço: Av. Hygino Muzzi Filho, 737
Bairro: Campus Universitário **CEP:** 17.525-900
UF: SP **Município:** MARILIA
Telefone: (14)3402-1346 **E-mail:** cep.marilia@unesp.br
