# Supplementary material for: Effect of different electrostimulation currents on female urinary incontinence: A protocol of a randomized controlled trial
Source: PLoS One. 2022 Dec 1;17(12):e0276722. doi: 10.1371/journal.pone.0276722 (PMC9714840; doi:10.1371/journal.pone.0276722)
Supplement: S2 Protocol — (PDF) [file pone.0276722.s003.pdf]

REPRESENTATED OPINION OF THE CEP

RESEARCH PROJECT DATA

Research Title: Effect of different electrical stimulation currents on the tibial nerve in women with urinary incontinence: Randomized Clinical Trial

Researcher: CRISTIANE RODRIGUES PEDRONI

Subject Area:

Version: 1

Case: 11479119.9.0000.5406

Proponent Institution: Faculty of Philosophy and Science / UNESP - Campus de Marília

Main Sponsor: Own financing
FOUNDATION FOR RESEARCH SUPPORT IN THE STATE OF SAO PAULO

OPINION DATA

Opinion Number: 3,272,572

Project presentation:

INTRODUCTION: Urinary Incontinence is defined as a complaint of any involuntary loss of urine. It can be classified into three main types, effort, urgency and mixed. Urinary Incontinence Urgency is characterized by involuntary loss of urine associated with urinary urgency, polyuria and nocturia. Some types of low-frequency electrostimulation have been used in clinical practice related to the treatment of urge urinary incontinence, but it is not yet known whether currents frequency currents can have effects similar to those of low frequency currents. PURPOSE: THE The aim of the study will be to verify the effects of the application of medium frequency currents in women who present urgent urinary incontinence and compare the effects of low currents frequency. METHOD: 105 volunteers aged between 18 and 80 years will participate in this research. complain of hyperactivity bladder (frequency increased urinary, nocturia or urge/incontinence. The ICIQ-SF, ICIQ-OAB, OAB-V8 and ISI questionnaires will be applied, as well as the diary micturition to assess symptoms of visual hyperactivity. They will be composed of 5 groups, which will be named according to the type of current that will be applied: G-TENSUrogynecology and G-TENS Conventional will be treated with TENS, G-Ausie will receive Ausie current, G-Interferential will receive interferential current and the G-High Voltage. All volunteers will undergo treatment with electro-stimulation between two to three times a week, totaling 20

sessions of 30 minutes each. Statistical processing will be performed using SPSS software, version 18.0 (SPSS Inc, Chicago, IL). Data will be presented as means and confidence interval a 95% for each variable. The Shapiro-Wilk test will be used to analyze data normality. For normal distribution parametric tests will be used to compare the means, if not, they will be used their respective non-parametric tests.

Research Objective:

Main goal

The aim of the study will be to verify the effects of applying medium frequency currents in women who have urge urinary incontinence.

Specific objectives

Check whether stimulation with medium frequency electrical current produces beneficial effects in the sense of decrease the frequency and volume of urinary loss in women with urge urinary incontinence, as well how to improve the quality of life of these women.

Compare the effects of medium frequency currents with the effects of low frequency currents used for the treatment of urge urinary incontinence.

Risk and Benefit Assessment:

Not applicable. There will be no burden for the participant and will receive assistance, if necessary, and guidelines that will help you in your health.

Research Comments and Considerations:

The research meets ethical criteria.

Considerations for Mandatory Submission Terms:

The terms requested by the Research Ethics Committee with beings were presented and analyzed humans.

Conclusions or Pending Issues and List of Inadequacies:

Approved.

Final Considerations at the discretion of the CEP:

The CEP of the FFC of UNESP of MARÍLIA, at an ordinary meeting of 04/17/2019, after accepting the opinion of the reporting member previously approved for this study and meeting all the provisions of the resolutions 466/2012, 510/2016 and complementary, as well as having approved the Consent Term Free and Clarified as well as all attachments included in the survey, resolves to APPROVE the research project Effect of different electrical stimulation currents on the tibial nerve in women with urinary incontinence: Randomized clinical trial.

Document Type	Archive	Post	Author	Situation
Basic Information from the project	PB_INFORMAÇÕES_BASICS_DO_PROJETO_1313429.pdf	04/09/2019 11:51:49		Accepted
Schedule	Schedule_ECR_corrigodo.docx	04/09/2019 11:51:30	CRISTIANE RODRIGUES PEDRONI	Accepted
Detailed project / Brochure Investigator	project_research_corrected.doc	04/09/2019 11:50:07	CRISTIANE RODRIGUES PEDRONI	Accepted
Declaration of Institution and Infrastructure	statement.pdf	03/29/2019 16:08:22	RAISSA ESCANDIUSI AVRAMIDIS	Accepted
TCLE / Terms of Assent / Justification of Absence	tcle.pdf	03/19/2019 21:03:15	RAISSA ESCANDIUSI AVRAMIDIS	Accepted
cover sheet	cover_sheet.pdf	03/19/2019 21:01:15	RAISSA ESCANDIUSI AVRAMIDIS	Accepted

Status of Opinion:

Approved

Needs Assessment from CONEP:

No

MARILIA, April 18, 2019

Signed by: CLAUDIO ROBERTO BROCANELLI

(Coordinator)
