# Supplementary material for: Effect of different electrostimulation currents on female urinary incontinence: A protocol of a randomized controlled trial
Source: PLoS One. 2022 Dec 1;17(12):e0276722. doi: 10.1371/journal.pone.0276722 (PMC9714840; doi:10.1371/journal.pone.0276722)
Supplement: S1 Appendix — (DOCX) [file pone.0276722.s004.docx]

**APPENDICE – Consent Form**

You are being invited to participate in a survey entitled "Effect of medium frequency currents in women with urgent urinary incontinence" which intends to study the effects of applying medium frequency currents in women with urge urinary incontinence.

You were selected to participate in this research because you have characteristics that are suitable for this study.

Step 1) This step consists of some questions about your personal life, history of illnesses and urination habits (how often you urinate). The questionnaires must be answered by you before the physical evaluation and will be part of a study with other women who will also answer the same questionnaires. The interview will last about 30 minutes.

Step 2) In this step, the physical examination will be performed, which consists of assessment of body measurements and vaginal muscles by digital touch. The physical examination will take about 15 minutes. All procedures are safe, but possible risks of the procedure are considered: discomfort and pain. The procedure is intended to help clarify how the pelvic floor muscles work.

Step 3) At the end of the treatment, you will be referred to a specialized sector for continuation of your treatment, if the symptoms have not stopped. After 1, 3 and 6 months we will contact you to verify the maintenance of the possible beneficial effects of this treatment.

Your benefit in participating in the research will be to receive treatment for urinary incontinence for urgent reasons and, if there is no satisfactory evolution at the end of the protocol, you will be guided and referred to the necessary treatment in a specialized sector. In addition, you will receive guidance on how to maintain your perineal health to avoid possible urine loss. The displacement for data collection will be on the same day that the treatment will start and end, therefore, it will not bring additional burden.

Please be aware that your participation in this study is voluntary and, even after you have given your consent to participate in the research, you can withdraw it at any time, without any prejudice to the continuity of your treatment. This Consent Form was prepared in 2 copies of equal content, 1 copy will be delivered to you signed and the other copy will be filed and kept by the researchers for a period of 5 years after the end of the research. If you have any additional questions, you can get in touch with the Research Ethics Committee through the telephones (14) 3402-1300, which operates from Monday to Friday from 8:00 am to 11:30 am and from 2:00 pm to 5:00 pm, at Avenida Higino Muzzi Filho, 737- Mirante , Marília-SP 17525-900. The main researcher is Dr Cristiane Rodrigues Pedroni who can be reached at Telephone: (14) 3402-1300 or by e-mail: [cristiane.pedroni@unesp.br](about:blank).

After all my doubts regarding this study have been resolved, I agree to participate voluntarily, being aware that all my data will be protected through the confidentiality that the researchers have undertaken. I am aware that the results of this study may be published in scientific journals, without my identity being revealed.

I:____________________________________________ID: _____________________

Phone:(___)__________Cell phone:(__)__________Address:____________________

I voluntarily agree to participate in the assessment and intervention procedures, which I have been informed.

Date: ____/____/_______ ______________________________________

Participant's Signature

Researcher Responsible for the data collection:

Name: ___________________________________ Phone: ______________________

Responsible Researcher: ______________________________
